# Well-Being Perception during COVID-19 Pandemic in Healthy Adolescents: From the Avatar Study

**DOI:** 10.3390/ijerph18126388

**Published:** 2021-06-12

**Authors:** Francesca Mastorci, Luca Bastiani, Gabriele Trivellini, Cristina Doveri, Anselmo Casu, Marta Pozzi, Irene Marinaro, Cristina Vassalle, Alessandro Pingitore

**Affiliations:** 1 National Research Council Institute of Clinical Physiology, 56124 Pisa, Italy; mastorcif@ifc.cnr.it (F.M.); luca.bastiani@ifc.cnr.it (L.B.); gabritri@ifc.cnr.it (G.T.); cristina.doveri@ifc.cnr.it (C.D.); webmaster@easywebmaster.eu (A.C.); irene.marinaro@ifc.cnr.it (I.M.); 2 Department of Addictions, ASFO—Azienda Sanitaria Friuli Occidentale, 33072 Pordenone, Italy; marta.pozzi@asfo.sanita.fvg.it; 3 Fondazione G. Monasterio, Regione Toscana, 56124 Pisa, Italy; cristina.vassalle@ftgm.it

**Keywords:** quarantine, COVID-19, health, well-being, adolescent, social context, lifestyle, mental skills, emotional reactivity

## Abstract

The COVID-19 pandemic provided an extraordinary and naturalistic context to observe young people’s psychosocial profiles and to study how a condition of environmental deprivation and lack of direct social contact, affects the well-being and health status of adolescents. The study explored whether the COVID-19 outbreak changes, in the short term, the acute well-being perception in adolescents, as measured by a Personalized Well-Being Index (PWBI) and the four components affecting health (i.e., lifestyle habits, social context, emotional status, mental skills), in a sample of early adolescent students. Data from 10 schools were collected on 1019 adolescents (males 48.3%, mean age 12.53 ± 1.25 y). Measurements were obtained at two time points, in September/October 2019, (baseline condition, BC) as part of the “A new purpose for promotion and eVAluation of healTh and well-being Among healthy teenageRs” (AVATAR) project and during the Italian Lockdown Phase (mid–late April 2020, LP), with the same students using an online questionnaire. During COVID-19 quarantine, adolescents showed a lower PWBI (*p* < 0.001) as compared to the BC. Considering the four health-related well-being components, lifestyle habits (*p* < 0.001), social context (*p* < 0.001), and emotional status (*p* < 0.001), showed significantly lower values during LP than BC. However, mental skills, in LP, displayed a significant increase as compared to BC (*p* < 0.001). In this study, we have provided data on the personalized well-being index and the different components affecting health in adolescents during the COVID-19 lockdown, showing a general decrease in well-being perception, expressed in lifestyle habits, social, and emotional components, demonstrating detrimental effects in the first phase of quarantine on adolescents’ psychosocial profiles. Our results shed new light on adolescence as a crucial period of risk behavior, especially when social support is lacking.

## 1. Introduction

The COVID-19 outbreak has profoundly changed the lives of many people across the world. Evidence obtained during SARS, MERS, and Ebola, have documented the negative long-term effects associated with quarantine and isolation, including anxiety, depression, sleep problems, and post-traumatic stress disorders, mostly in patients and health-care workers [1,2]. However, not much is known about the acute effects on ordinary citizens, such as children and adolescents, representing an important gap for research [3,4]. These, although less likely to be infected with COVID-19, are not unresponsive to the psychosocial effects of the pandemic [5]. In fact, in adults, the adverse outcomes of the pandemic are mainly related to fear of being infected, job loss, and stigma [6,7,8], whereas in adolescents, they are primarily due to the closure of schools and separation from friends, and sometimes, from family, although data about pandemic effects is scarce and obtained in other home confinement conditions [3,9]. Only one study, in children, evaluated the psychological impact of quarantine as a predictor of long-lasting post-traumatic stress symptoms, reporting four times higher probability of developing it than in those who were not in quarantine [3]. Adolescence, in normal conditions, is a period of marked social changes, in which social relationships are responsible for the development of self-regulating senses of identity and emotional reactivity, promoting healthy well-being [10]. In addition, adolescence is also a “sensitive window” of brain development and refinement, in which the social environment can exercise a strong impact on resilience or vulnerability [11]. In fact, on one side, adolescence is considered a time of good health when disease burden is low, and on the other, during this time there is an increased susceptibility to the onset of mental diseases [12]. Actually, the concurrent psychosocial alterations due to COVID-19 can seriously leave a negative impact on adolescents’ health, in both acute and long-term.

The hypothesis of the present study is whether social relationships help promote healthy behaviors, ensuring that adolescents are exposed to environmental enrichment; social deprivation, and therefore quarantine, can on the contrary modify resilience, empowerment and well-being, thus becoming a moment of emotionality, cognitive and social reset. A personalized well-being index (PWBI) was previously developed and already tested in healthy adolescents; on the basis of the relationship among the different weights of the variables of four well-being components, that is, emotional status, lifestyle habits, social context and mental skills [13]. The current study is aimed at investigating whether the COVID-19 outbreak changes, in the short-term, the well-being perception in adolescents, as measured by PWBI in a sample of early adolescent students.

## 2. Materials and Methods

### 2.1. Participants and Study Design

The survey, “A new purpose for promotion and eVAluation of healTh and well-being Among healthy teenageRs”(AVATAR) COVID-19 was conducted during phase 1 of the Italian lockdown (mid–late April 2020), using an online questionnaire, as part of the AVATAR project, in order to develop a new tool to assess lifestyle habits, social context, emotional status, and mental skills in adolescents, and to define an integrated index of the best indicators of well-being [13,14,15]. During the COVID-19 pandemic, ten junior high schools participated in the AVATAR COVID-19 study, a section of the AVATAR project.

Schools that participated in the survey were located in Central and Northern Italy, mainly in Tuscany (seven schools), one in Liguria, and two in Friuli Venezia Giulia. The choice of schools depended on their voluntary participation in the project.

According to the AVATAR approach, data were usually collected at the beginning and at the end of the school year, in order to allow teachers to evaluate the effectiveness of educational strategies defined on the basis of the identified needs. During the school year 2019/2020, in September/October, 3458 students aged between 10 and 14 years were monitored, and 1019 of these completed the questionnaires during the lockdown (April 2020). Therefore, the final population consisted of 1019 adolescents with data acquired in standard conditions (at the beginning of school year) and during COVID-19 quarantine.

Adolescent students were enrolled according to the following inclusion criteria: age 10–14 years, absence of neuropsychiatric or other diseases, signed informed consent, and filling of the entire proposed questionnaires.

In every school class, all the adolescents filled out the questionnaire, and, if they were not eligible due to exclusion criteria, they were excluded from the study retrospectively. All students filled out questionnaires in two different phases: Baseline Conditions (BC), at the beginning of the school year, and during the Lockdown Phase (LP).

Participants were previously instructed on how to fill out the questionnaires and how to conduct the tests. All tests, during the first monitoring, were conducted during participants’ computer lessons in school time, while during quarantine, tests were completed during distance learning in the presence of a teacher. No incentive was provided to adolescents or parents. A research assistant was available to provide information and technical support to complete questionnaires in both conditions.

### 2.2. Ethics

The experimental protocol was approved by the internal ethics committee of each participating school, joining Rete Ulisse, in accordance with Italian law. In addition, all parents or legal guardians gave informed consent, and authorized researchers to use their data in accordance with Italian law. All procedures performed in the study were in accordance with the ethical standards of the institutional, national, or both, research committees and with the 1964 Helsinki declaration and its later amendments or comparable ethical standards. The AVATAR project has been accepted by the Regional Pediatric Ethics Committee (Azienda Ospedaliero Universitaria Meyer) (code 166/2018).

### 2.3. Procedures

Data were collected with AVATAR Web-tool [14]. A socio-demographic data record was used to collect information on gender, age, and schooling. The Italian version of KIDSCREEN-52 was used to assess health-related quality of life [16,17]. KIDSCREEN is a self-report questionnaire designed to address health-related quality of life, aimed at monitoring and measuring the personal experiences in children and adolescents regarding their perception of health status and well-being. The questionnaire, describing physical, psychological, mental, social, and functional aspects of well-being, consists of 52 items grouped into 10 dimensions (physical well-being, psychological wellbeing, moods and emotions, self-perception, autonomy, parent relations and home life, social support and peers, school environment, social acceptance (bullying), and financial resources). Some sample items, “In general, how would you say your health is?” for the physical well-being dimension; “Have you felt satisfied with your life?” for moods and emotions; “Have you been happy with the way you are?” for self-perception. Cronbach’s alphas range from 0.77 to 0.89 for the dimensions of the 52-item version. KIDSCREEN questionnaires were psychometrically tested using data obtained in a multicenter European study which included a sample of 22,827 children recruited in 13 countries [18].

Dietary habits were evaluated using the Mediterranean Diet Quality Index for children and adolescents (KIDMED) [19]. The KIDMED index was based on principles sustaining Mediterranean dietary patterns as well as those that undermine it (for example, “Every day I eat fruit or freshly squeezed fruit juice”, “Regularly once a day would consume fresh and cooked vegetables”, and “I eat pasta and rice almost every day (5 or more per week)”). The index ranged from 0 to 12, and consisted of a self-administered 16-question test. The validity of the KIDMED index is demonstrated by the evidence that a higher score is associated with the expected patterns of food and nutrient intake representative of a good quality diet. (Cronbach’s alpha = 0.79, 95% CI: 0.71–0.77). Physical activity levels were assessed using the Physical Activity Questionnaire for Older Children (PAQ-C). The questionnaire provides a general measure of physical activity for 8- to 20-year-olds. The PAQ-C is a self-administrated questionnaire consisting of nine items rated on a five-point scale. (e.g., “In the last 7 days, during your physical education (PE) classes, how often were you very active (playing hard, running, jumping, throwing)?”, “In the last 7 days, on how many evenings did you do sports, dance, or play games in which you were very active?”, “On the last weekend, how many times did you do sports, dance, or play games in which you were very active?”). The average of the items is used to create the final PAQ summary score, a higher score indicates more active children/adolescents. Previous studies have supported the validity of the PAQ instrument for assessing general levels of physical activity. Validation studies have found the PAQ-C to be highly reliable (Cronbach alphas ranged from 0.72 to 0.88) [20]. The mental skills linked to school engagement has been estimated through questions concerning the scholastic achievement in language and literature, language acquisition, science, and artistic and creativity [15].

### 2.4. AVATAR Approach

Approach here is defined as focusing on the integration of four components of health-related well-being (lifestyle habits (LH); emotional status (ES); social context (SC); and mental skills (MS)), as perceived by adolescent [15]. The four components were obtained from the different variables analyzed by the questionnaires according to a structural model previously described in Mastorci and colleagues [15].

In detail, the path analysis technique used measures the extent to which the model fits a data set and allows testing of interrelationships between several variables simultaneously. The confirmatory factor analysis was used to test an overall measurement model that included five correlated latent variables. Overall model fit was assessed using different statistics. First, a chi-square analysis was used. The other indices were the root mean square error of approximation (RMSEA) (values between 0.05 and 0.08 indicate acceptable fit, and values <0.05 a good fit), comparative fit index (CFI) (values >0.90 indicate reasonable fit, >0.95 good fit), and standardized root mean square residual (SRMR) (values <0.10 indicate good fit). The measurement model was first tested to ensure that each of the observed variables was a sufficient indicator of the hypothesized latent variables.

In addition, from the sum of the four components, we obtained a personalized well-being index (PWBI), ranging from 0 to 100, according to the AVATAR model as reported before [13].

As described by Mastorci and colleagues, PWBI was obtained by the integration of four components of health-related well-being (lifestyle habits (LH); emotional status (ES); social context (SC); and mental skills (MS)), as perceived by adolescents [13]. Both computed and user-provided data, obtained from the platform, can be mapped onto a specific axis of Σ space. Consequently, PWBI implementation is referred to the estimation of the mappings between the predefined subareas (ΣL, ΣS, ΣE, and Σ MS) and the four axes (L, S, E, and MS):

Lifestyle habits (L): ΣL → ΩL
Social context (S): ΣS → ΩS
Emotional status (E): ΣE → ΩE
Mental skills (MS): → Σ MS Ω MS

Each of these causal relationships is modelled by a linear equation with the cause(s) as independent variable(s). Structural equation models (SEMs), widely used in psychometry and behavioral sciences, were used to implement the association phase (Henríquez et al., 2017). This choice was motivated by the moderate complexity of a linear SEM, which despite a possible negative influence on the estimation accuracy, is advantageous with respect to overfitting issues.

In this view, estimation of the four components of the PWBI is based on the relation process and PWBI was hypothesized to cause changes in lifestyle habits (L), social context (S), emotional status (E), and mental skills (MS).

Thanks to parameter estimates extracted from the SEM model (factor loadings and residual variance), an equation was defined to compute the (rescaled) personalized well-being index (PWBI) of each student. Subsequently, in order to implement PWBI in the AVATAR web-tool for teachers and parents, the PWBI score was rescaled to a range of 0–100, using the following formula:

100 × (PWBI − min)/(max − min).

The result of this equation was used as the PWBI factor score for each student, a continuous trait that summarizes the common components of PWBI.

The equations are shown below.

PWBI = 0.954 × Lifestyle habits (L) + 0.972 × Social context (S) + 0.949 × Emotional status (E) + −0.141 × Mental skills (MS).

### 2.5. Statistical Analysis

Statistical data analyses were performed using SPSS (Version 22.0. Armonk, NY, USA: IBM Corp). Data are presented as mean ± SD. Alpha was set at 0.05 and 2-sided *p*-values were reported. The Shapiro–Wilk test was used to test for normality of data. Changes in variables, four components, PWBI, and percentage changes in the definition of the PWBI in baseline condition as compared to lockdown phase were analyzed by Student’s paired *t*-test.

## 3. Results

### 3.1. Acute Effect of COVID-19 Pandemic on Different Psychosocial Variables Composing the PWBI

Descriptive data on psychosocial variables comprising the PWBI in the study population, in BC and LP, are presented in Table 1. Data on the KIDSCREEN-52 dimension are calculated as the mean T-scores according to KIDSCREEN Group [17].

During COVID-19 quarantine, adolescents showed on average a lower perception in the psychological well-being (*p* < 0.001), physical well-being dimensions (*p* < 0.001), a poorer mood/emotion (*p* < 0.05), as well as a lower autonomy (*p* < 0.001), understood as the opportunity to create his/her social and leisure time. In social context assessment, adolescents reported lower values in their relationships with peers (*p* < 0.001), but also exhibiting a higher perception of social acceptance (*p* < 0.001) during quarantine. For lifestyle, adolescents developed higher adherence to the Mediterranean diet (*p* < 0.001) as compared to baseline conditions. In the mental skills component, school performance in language and literature **(***p* < 0.001) and language acquisition (*p* < 0.001), during lockdown phase showed lower scores, while school performance perception in scientific and artistic fields revealed higher values (*p* < 0.001) than the pre-COVID-19 period.

### 3.2. Personalized Well-Being Index and Health-Related Well-Being Components

The total population analyzed in both conditions, BC and LP, was composed of 1019 students (males 48.3%), mean age 12.53 ± 1.25 y. During COVID-19 quarantine, adolescents showed a lower PWBI as compared to the baseline conditions (56.72 ± 9.34 vs. 53.89 ± 10.44; *p* < 0.001).

When we considered the health-related well-being components (Table 2), lifestyle habits (*p* < 0.001), social context (*p* < 0.001), and emotional status (*p* < 0.001), showed significantly lower scores during quarantine than baseline values. However, mental skills, for the period of distance learning, displayed a significant increase compared to baseline settings (*p* < 0.001). Considering the four areas as percentages of the total PWBI (the sum of the four areas gives the index), a significant difference is observed for all components during the lockdown compared to the baseline condition (Table 3).

## 4. Discussion

The present study explores, for the first time, the short-term effects of COVID-19 quarantine on four health-related well-being components (lifestyle habits, social context, emotional status, and mental skills) and PWBI in adolescents, that is an integrated score of above-mentioned dimensions. The main results can be summarized according to two perspectives: (1) considering the single variables within each component and (2) analyzing the four health-related well-being components forming the PWBI. Firstly, quarantine induces a lower perception in the psychological and physical well-being, in mood/emotion, autonomy, relationship with peers, as well as the perception of bullying, while the lockdown period seems to encourage healthy behaviors in terms of higher adherence to the Mediterranean diet and creativity. Secondly, according to an integrated approach between the variables and the components, the key findings can be summarized in the following points: (i) during the lockdown phase, adolescents showed a lower well-being perception as shown by the decreased PWBI; (ii) the four areas, composing PWBI, although maintaining the same order of importance, changed significantly in percentage terms, increasing their contribute to the emotional status during quarantine; (iii) lifestyle habits, social context, and emotional status, in absolute values, decreased during quarantine compared to baseline conditions, whereas mental skills were enhanced during the COVID-19 outbreak.

The circumstances related to the COVID-19 pandemic provided an extraordinary and naturalistic context to observe young people’s psychosocial profiles, and to study how a condition of environmental deprivation and lack of direct social contact, affects adolescents’ well-being. Before COVID-19, the evidence on the effects of isolation came primarily from animal models [21,22,23]. Human studies were mainly focused on adults, while there are very few comparable studies on adolescents regarding their quarantine experiences [7,8]. In adolescents, on the other hand, studies have predominantly pointed out that the nature, quality, and complexity of social connections, and not social deprivation, are positively correlated with affective and mentalizing systems [24]. On the contrary, a lack of social connection, such as quarantine, can have detrimental effects on the brain. In fact, neuroimaging evidences have shown that individuals who report a lack of relations display problems in processing social input [25].

There are few studies that have evaluated the effects of quarantine in adolescents [26,27,28].

In comparison to previous studies, we enrolled healthy adolescents aged between 10 and 14 years, who filled questionnaires, the first one at school and the second one at home during confinement [3,9]. Moreover, we administered questionnaires assessing self-perception of well-being without any kind of clinical implication. This means that we maintained our evaluation in a preclinical condition, assessing the difficulties and the issues raised with quarantine, potentially leading, in the long-term, to post-traumatic stress disorder or other clinical problems. In the present study, the four PWBI components were also individually assessed in order to ascertain their changes during COVID-19 quarantine [13,29]. Thanks to this index, it is possible to identify, under stressful conditions such as quarantine, the strength and the fragile characteristics of each adolescent to potentiate the first ones and to change or improve the others through the application of personalized educational programs.

The results showed that PWBI significantly fell during COVID-19 quarantine and its breakdown corresponded to a lower score in three of four components, which are lifestyle, social context, and emotional status. The basal data confirmed the results of our previous work, which highlighted the preponderant role of social context and the lack of involvement of mental skills in the perception of well-being [13].

Our data seem to confirm what has recently been hypothesized from Italian, Spanish, and Chinese studies, that suggests significant emotional and behavioral changes during COVID-19 quarantine in adolescents [28,30]. In fact, emotional status of our sample, during isolation, in terms of self-perception and psychological well-being, compared to the baseline values, was significantly reduced in absolute values. However, when we considered the percentage in which the four areas made up the PWBI, during quarantine, emotional dimension increased to indicate that in these conditions, the psychological state, although reduced in absolute values, acquired more importance. In other words, if in basal conditions the perception of well-being was much more linked to the social context, during quarantine, an increase in the psychological component is overlapped to a reduction in the role of the social context.

Interestingly, during the closing period of schools and distance learning, we observed an improvement in the cognitive skills compared to baseline condition. This increase in mental abilities could be explained by the fact that adolescents usually live under such intense stress conditions until they develop burnout, possibly related to school, routine, friendships, and other responsibilities [31]. Indeed, this data could be in line with previous studies showing that difficult situations, sometimes, through resilience mechanisms and empowerment, can have positive effects on cognitive aspects and in general on health status [32,33]. Data obtained during natural disasters, for example, have shown that resilience depends on adequate communication and preparedness, good social support, and capacity to adapt and cope with traumatic events [33].

In addition, in terms of lifestyle, our results are in agreement with the notion that asserts that staying at home, with limitation of outdoors and in-gym physical activity, compromises the perception of heaving healthy habits. In our case, overall behaviors linked to lifestyle habits, such as spare time, the perception of health, and economic well-being, and not only diet and physical activity are reduced [15]. However, a recent Italian study, aimed at exploring the impact of the COVID-19 pandemic on eating habits and lifestyle changes among Italian adolescents, demonstrated an improvement in lifestyle, with a reduction in tobacco consumption and a higher adherence to the Mediterranean diet [34].

The main limitation of the present study is represented by a self-reported questionnaire and the different places of monitoring. In fact, in baseline, the questionnaires were completed during a school class, and it is possible that the school classroom environment may have biased the students’ responses, especially for items related to the school class environment; while during quarantine, although questionnaires were completed during distance learning in the presence of a teacher, they were conducted in the home context. However, our web survey was the same one used in other protocols by our group [13,15,29]. Furthermore, the study group consisted of young adolescents monitored in a sample of schools and cannot be considered representative of all adolescents.

A strength of our study was represented by the fact that the survey was conducted quickly in the most critical period of the pandemic in Italy, after one month from the start of the lockdown.

## 5. Conclusions

In this study, we showed a general decreased in well-being perception in the acute phase of the COVID-19 lockdown. Quarantined adolescents are at high risk of developing higher risk psychological health-related challenges, probably in both the acute and chronic periods. These findings suggest the need to integrate psychosocial health care into the planning and implementation of preventive strategies for quarantine measures. In particular, schools and health institutions should implement guidelines for the psychosocial support of adolescents already in the early stages of the health emergency, in order to avoid the long-term impact of such stress on mental health. It is pivotal that educational institutions, health authorities, and parents work together to reduce adolescents’ emotional distress, fear, and anxiety through communication and facilitating professional counseling to address stressors and their possible countermeasures. Therefore, as the COVID-19 pandemic is still ongoing, future longitudinal studies to explore the relationship between psychological dimensions and social context and the effects of school closure and other social distancing practices in adolescence, are urgently needed to shed light on the pathways to resilience or vulnerability mechanisms for the development of psychopathology in the short and in the long-term.

## Figures and Tables

**Table 1:** Score of KIDSCREEN-52 domains, lifestyle habits, and school performance, divided by four components of health-related well-being in study sample in baseline conditions (BC) and during lockdown phase (LP).

	Variables	BC (*n* = 1019)	LP (*n* = 1019)	*p*-Value
Lifestyle habits	Physical wellbeing	46.95 ± 6.67	43.72 ± 6.97	<0.001
	Autonomy	47.12 ± 9.91	42.36 ± 8.61	<0.001
	KIDMED	5.99 ± 2.6	6.46 ± 2.48	<0.001
	PAQ-C	2.65 ± 0.69	2.65 ± 0.76	0.797
	Financial resources	51.08 ± 9.32	50.59 ± 10.37	0.095
Emotional status	Psychological wellbeing	50.23 ± 9.37	48.87 ± 9.83	<0.001
	Mood/Emotion	48.62 ± 9.9	48.03 ± 9.82	<0.05
	Self-perception	52.92 ± 10.62	52.89 ± 11.19	0.908
Social context	Parent relationship	51.15 ± 9.98	50.78 ± 10.13	0.215
	Peers	50.58 ± 10.12	41.49 ± 12.67	<0.001
	School environment	49.95 ± 8.67	50.38 ± 8.71	0.089
	Social acceptance (Bullying)	50.28 ± 10.41	52.41 ± 9.33	<0.001
Mental skills	School performance-Language and Literature	37.04 ± 3.68	33.6 ± 4.1	<0.001
	School performance-Science	34.51 ± 4.3	37.93 ± 3.84	<0.001
	School performance- Language acquisition	26.17 ± 3.05	23.94 ± 3.26	<0.001
	School performance- Artistic and creativity	23.27 ± 3.47	25.54 ± 3	<0.001

Data given as mean ± SD (95% CI). Data on the KIDSCREEN-52 dimension are calculated as the mean T-scores according to KIDSCREEN group. ns: not significant. *p*-values were calculated via Student’s paired *t*-test.

**Table 2:** Four components of health-related well-being in the study sample in baseline conditions (BC) and during lockdown phase (LP).

Variables	BC (*n* = 1019)	LP (*n* = 1019)	*p*-Value
Lifestyle habits	13.75 ± 2.88	11.93 ± 3.31	<0.001
Emotional status	19.54 ± 3.96	18.93 ± 4.94	<0.001
Social context	21.77 ± 3.72	20.09 ± 4.89	<0.001
Mental skills	0.3 ± 0.18	0.4 ± 0.37	<0.001

Data presented are mean value ± SD.

**Table 3:** Percentage changes in the definition of the personalized well-being index (PWBI) in baseline condition (BC) compared to lockdown phase (LP).

Variables	BC (*n* = 1019)	LP (*n* = 1019)	*p*-Value
Lifestyle habits	25%	23%	<0.001
Emotional status	35%	37%	<0.001
Social context	40%	39%	<0.001
Mental skills	0%	1%	<0.001

Data presented are % values.

## Data Availability

The datasets used, analyzed, or both, during the current study are available from the corresponding author on reasonable request.
